# Supplementary material for: Complex dynamics in a synchronized cell-free genetic clock
Source: Nat Commun. 2022 May 23;13:2852. doi: 10.1038/s41467-022-30478-2 (PMC9126873; doi:10.1038/s41467-022-30478-2)
Supplement: Supplementary file 1 — Supplementary Information [file 41467_2022_30478_MOESM1_ESM.pdf]

Supplementary Information for
Complex Dynamics in a Synchronized Cell-Free Genetic Clock

Lukas Aufinger, Johann Brenner, and Friedrich C. Simmel

Physics Department E14, D-85748 Garching, Germany

(Dated: May 4, 2022)

Contents

- Derivation of equation (5)
- Supplementary Figures 1-4

A. Derivation of equation (5)

To find an expression for the continuous dilution rate δ in terms of the semi-continuous refresh ratio R, we start by diluting a reagent with concentration c and initial concentration c_0. In the continuous case this is described by the simple ODE

$$\frac{dc}{dt} = -\delta \cdot c \ , \tag{1}$$

with the solution

$$c(t) = c_0 \cdot e^{-\delta t} \ . \tag{2}$$

In the semi-continuous case the concentration after u cycles of duration t_{int} is

$$c_u = c_0 \cdot (1 - R)^u \ . \tag{3}$$

We can now set $t = u \cdot t_{int}$ and equate $c_u = c(u \cdot t_{int})$. Inserting and rearranging then gives equation (5)

$$c_0 \cdot (1 - R)^u = c_0 \cdot e^{-\delta t_{int} \cdot u} \ , \tag{4}$$

$$1 - R = e^{-\delta t_{int}} \ , \tag{5}$$

$$\delta = -\frac{\ln(1 - R)}{t_{int}} \ . \tag{6}$$

B. Supplementary Figures

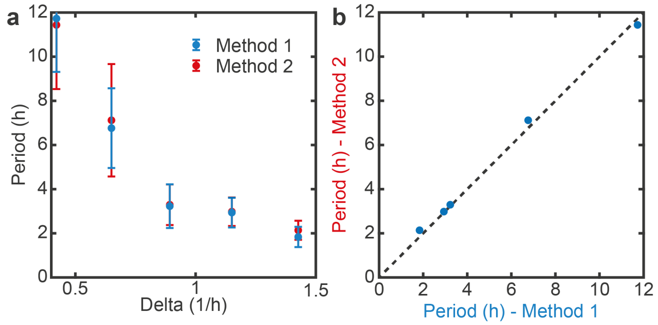

Supplementary Figure 1. Comparison of two alternative methods to determine the period of the free running oscillator in Fig. 3. For method 1, we used the 1st maximum of the spline interpolated autocorrelation function (ACF). For method 2, we excluded the initial, transient maximum from the time traces and then fitted a decaying cosine ($f(t; T, \tau) = e^{-t/\tau} \cdot \cos(2\pi t/T)$ with period T and de-correlation time τ) to the ACF. a) Shows the same plot as in Fig. 3c, with the results of both methods. Data shows means $\pm$ SD of $N = 2$ (first and last point) or $N = 4$ (other points) technical replicates. As time traces with periods > 6 h only contain 2-3 maxima, we additionally accounted for a systematic measurement uncertainty that scales inversely with the number of maxima. b) Direct comparison of the periods estimated with both methods.

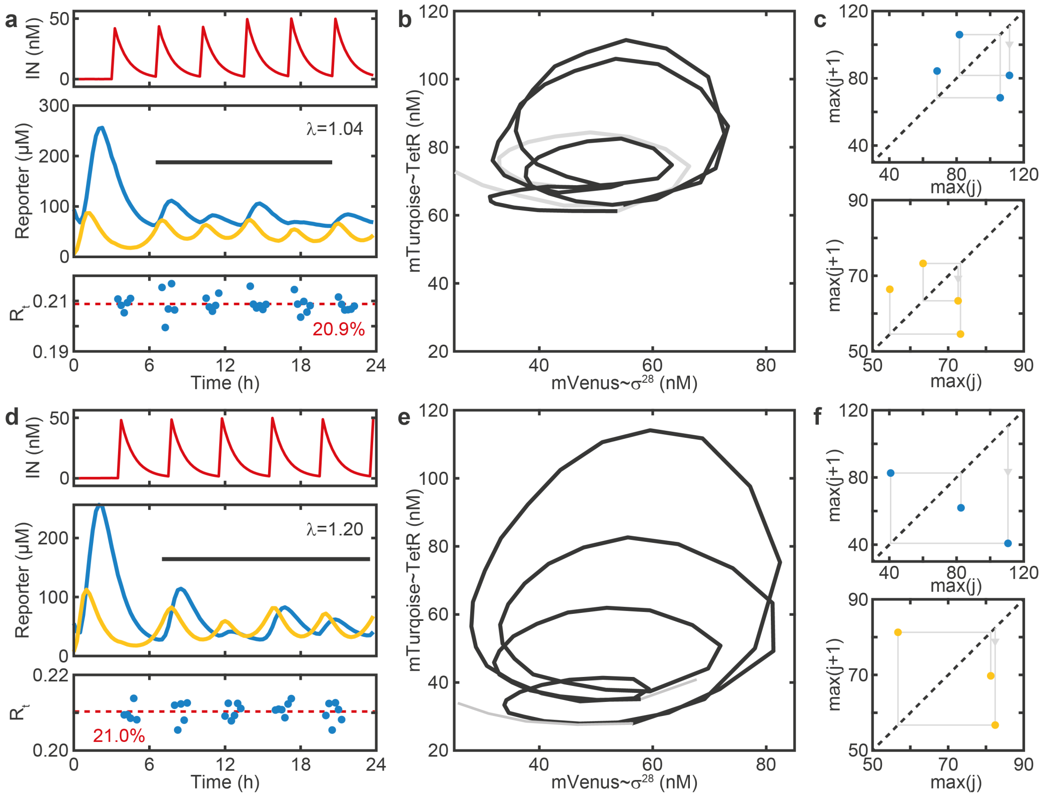

Supplementary Figure 2. Extended data of a TetR forcing experiment that was run for 24 h, at a-c) $\lambda = 1.04$ and d-f) $\lambda = 1.20$. a,d) Time traces of input and output signals, as well as instantaneous refresh ratio R_t. b,e) Corresponding phase portraits and c,f) maximum return maps. While the recorded time traces are too short to unequivocally prove period doubling, we can observe the same signatures that were observed in the longer experiments (Figs. 5 & 6 of the main text).

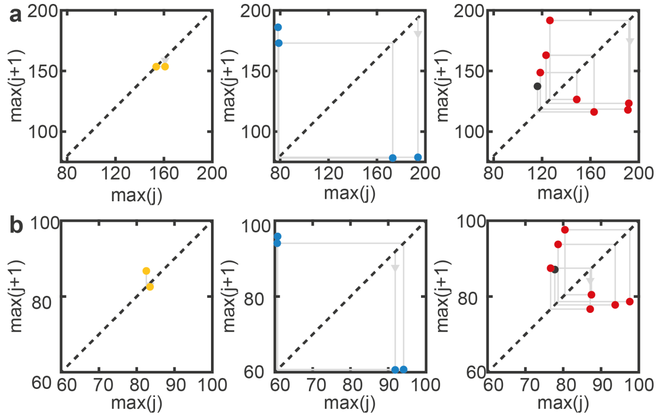

Supplementary Figure 3. Maximum return maps of the data presented in Fig. 6b for the a) mTurquoise∼TetR and b) mVenus∼ σ^{28} signal. From left to right the plots show a 1-cycle ($\lambda = 1.48$), a 2-cycle ($\lambda = 1.05$), and a 4-cycle ($\lambda = 1.20$).

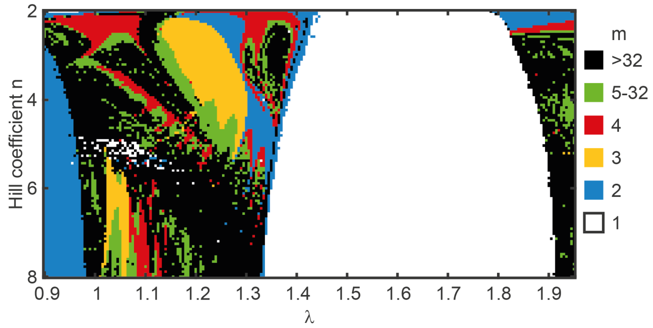

Supplementary Figure 4. Same plot as Fig. 7, showing the rotation number m for different values of the Hill coefficient n and λ, but with aTc as the external signal. Parameter values are the same as in Fig. 7. Activation with aTc was modeled with a strong binding reaction ($k_+ = 1000$ nM^{-1}s^{-1}, $k_- = 1$ s^{-1}) and input amplitude $A_{in} = 10$ nM.
