# Supplementary material for: Complex dynamics in a synchronized cell-free genetic clock
Source: Nat Commun. 2022 May 23;13:2852. doi: 10.1038/s41467-022-30478-2 (PMC9126873; doi:10.1038/s41467-022-30478-2)
Supplement: Supplementary file 3 — Description of Additional Supplementary Files [file 41467_2022_30478_MOESM3_ESM.pdf]

Title: Supplementary Data 1

Description: Contains a table with the DNA sequences of the plasmids and sequencing/colony PCR primers used in this study.
